# Genomics data of *L. borgpetersenii* strain HP364 and *L. weilii* strain SC295 isolated from rodents captured from human leptospirosis outbreak areas in Selangor, Malaysia

**DOI:** 10.1128/MRA.00859-23

**Published:** 2023-11-14

**Authors:** Vasantha Kumari Neela, Noraini Philip, Pappitha Raja, Zamberi Sekawi

**Affiliations:** 1 Department of Medical Microbiology, Faculty of Medicine and Health Sciences, Universiti Putra Malaysia, Selangor, Malaysia; 2 School of Biological Sciences, Universiti Sains Malaysia, Pulau Pinang, Malaysia; 3 Foundation Programme Trainee, University of Nottingham, University Park, Nottingham, United Kingdom; University of Arizona, Tucson, Arizona, USA

**Keywords:** *Leptospira borgpetersenii*, *Leptospira weilii*, whole genome sequencing, rodents

## Abstract

We report complete genome sequences of two *Leptospira* isolates, *Leptospira borgpetersenii* strain HP364 and *Leptospira weilii* strain SC295. The genome sizes of *L. borgpetersenii* strain HP364 and *L. weilii* strain SC295 were 3,874,738 bp and 4,063,712 bp, respectively. Both genomes have been deposited in NCBI GenBank.

## ANNOUNCEMENT

The isolation of *Leptospira*, the etiologic agent of leptospirosis, from the reservoir hosts is important for species identification and virulence analysis. The data reported in this article are the genomics data of the whole genome sequencing of *Leptospira borgpetersenii* strain HP364 (*L. borgpetersenii* strain HP364) and *Leptospira weilii* strain SC295 (*L. weilii* strain SC295) (Table 1). Both *Leptospira* species were isolated from the kidneys of rodents captured randomly from the human leptospirosis suspected area, in forest environments (1). The kidney was aseptically harvested; washed with 2 mL of sterile phosphate-buffered saline solution; transferred into a 15 mL Falcon tube containing 5 mL of liquid Ellinghausen, McCullough, Johnson, and Harris (EMJH) medium supplemented with *Leptospira* enrichment medium, rabbit and calf serum, and 5-fluorouracil; and macerated using a sterile glass rod. The supernatant (0.5 mL) was then inoculated into fresh EMJH medium and incubated at 28°C for growth of leptospires (3 months of incubation). The culture was filtered and sub-cultured in EMJH medium (28°C) until pure *Leptospira* culture was obtained.

**TABLE 1:** The information of genomics data of *L. borgpetersenii* strain HP364 and *L. weilii* strain SC295

Parameter	*L. borgpetersenii* strain HP364	*L. weilii* strain SC295
Species of rodents	*Rattus rattus*	*Sundamys muelleri*
Location	Hulu Perdik, Selangor, Malaysia	Sungai Congkak, Hulu Langat, Selangor, Malaysia
Year of isolation	2017	2016
Sequencing coverage	262×	206×
Total number of paired reads	3,361,111	2,773,480
Genome size	3,874,738 bp	4,063,712 bp
GC content	40.1%	40.8%
Number of contigs	122	220
N50	182,549 bp	128,583 bp
Number of subsystems	224	225
Number of coding sequences	4,234	4,578
Number of RNAs	40	39
BioSample accession number	SAMN35669191	SAMN35682233
BioProject accession number	PRJNA981232	PRJNA981655
Genome accession number	JASUZS000000000	JASUZT000000000
SRA accession numbers	SRX21668559 PRJNA981232	SRX21668273 PRJNA981655

The genomic DNA of pure cultures *L. borgpetersenii* strain HP364 and *L. weilii* strain SC295 was extracted using DNeasy Blood & Tissue kits (Qiagen, Germany) according to the manufacturer’s instructions. The genomic DNA was sent for high-throughput DNA Sequencing at the Genome Institute of Singapore, Next Generation Sequencing Facility (IGP-NGSP; A*STAR, Singapore). The DNA library was prepared using the IGP-NGSP Illumina Library preparation (Qiagen QIAseq Fx DNA Library Kit protocol). Sequencing was performed using the 2 × 151 bp paired-end format on the Miseq platform (Illumina, San Diego, CA). FastQC (v0.11.8) was used to assess the quality of the raw sequences. The adapters and sequences read with less than 50 bp were trimmed and removed using BBDuk.sh (BBTools v38.43) (2). SPAdes (v0.4.6) (3) was used to *de novo* assemble the quality-filtered sequences. The draft genomes of *L. borgpetersenii* strain HP364 and *L. weilii* strain SC295 were annotated by the Rapid Annotation of microbial genomes using Subsystem Technology (v2.0) for subsystem classification and functional annotation (4) and NCBI Prokaryotic Genome Annotation Pipeline (v6.5) for generation of the publicly available annotations (5–7). Default parameters were used for all software unless further specified.

The availability of these genomics data could provide fundamental knowledge and insight toward understanding the virulence and microbial properties of *Leptospira* isolates in Malaysia.

## Data Availability

The genome sequences have been deposited in NCBI GenBank under the accession numbers JASUZS000000000 (*L. borgpetersenii* strain HP364) and JASUZT000000000 (*L. weilii* strain SC295). Sequencing reads are available in Sequence Read Archive (SRA) under accession numbers PRJNA981232 (*L. borgpetersenii* strain HP364) and PRJNA981655 (*L. weilii* strain SC295).
